# The Monetary Burden of Cystic Echinococcosis in Iran

**DOI:** 10.1371/journal.pntd.0001915

**Published:** 2012-11-29

**Authors:** Majid Fasihi Harandi, Christine M. Budke, Sima Rostami

**Affiliations:** 1 Research Center for Modeling in Health, Kerman University of Medical Sciences, Kerman, Iran; 2 Department of Parasitology, School of Medicine, Kerman University of Medical Sciences, Kerman, Iran; 3 Department of Veterinary Integrative Biosciences, College of Veterinary and Biomedical Sciences, Texas A&M University, College Station, Texas, United States of America; University of Zurich, Switzerland

## Abstract

Cystic echinococcosis (CE) is a globally distributed parasitic infection of humans and livestock. The disease is of significant medical and economic importance in many developing countries, including Iran. However, the socioeconomic impact of the disease, in most endemic countries, is not fully understood. The purpose of the present study was to determine the monetary burden of CE in Iran. Epidemiological data, including prevalence and incidence of CE in humans and animals, were obtained from regional hospitals, the scientific literature, and official government reports. Economic data relating to human and animal disease, including cost of treatment, productivity losses, and livestock production losses were obtained from official national and international datasets. Monte Carlo simulation methods were used to represent uncertainty in input parameters. Mean number of surgical CE cases per year for 2000–2009 was estimated at 1,295. The number of asymptomatic individuals living in the country was estimated at 635,232 (95% Credible Interval, CI 149,466–1,120,998). The overall annual cost of CE in Iran was estimated at US$232.3 million (95% CI US$103.1–397.8 million), including both direct and indirect costs. The cost associated with human CE was estimated at US$93.39 million (95% CI US$6.1–222.7 million) and the annual cost associated with CE in livestock was estimated at US$132 million (95% CI US$61.8–246.5 million). The cost per surgical human case was estimated at US$1,539. CE has a considerable economic impact on Iran, with the cost of the disease approximated at 0.03% of the country's gross domestic product. Establishment of a CE surveillance system and implementation of a control program are necessary to reduce the economic burden of CE on the country. Cost-benefit analysis of different control programs is recommended, incorporating present knowledge of the economic losses due to CE in Iran.

## Author Summary

Cystic echinococcosis (CE), caused by the tapeworm *Echinococcus granulosus*, is a zoonotic infection that occurs worldwide. The adult parasite resides in the small intestines of dogs and the cyst form can develop in the liver and lungs of both humans and livestock. CE causes medical, veterinary, and economic losses in endemic areas. However, data on the economic consequences of CE are lacking. The present study estimated the monetary burden of CE in Iran. We used epidemiological and economic information to estimate direct and indirect costs of human and livestock CE in the country. Costs associated with human CE included the costs of surgery and hospital services in addition to lost wages due to work absenteeism during hospitalization and recovery. Costs associated with CE in livestock included losses due to condemnation of livers and lungs during carcass inspections, decreased carcass weight, reproductive losses, and reductions in milk and other animal products. We estimated the overall annual cost of CE in Iran at US$232.25 million, with the cost of the disease estimated to be approximately 0.03% of the country's gross domestic product. Implementation of a control program is necessary to reduce the economic burden of CE on Iran.

## Introduction

Cystic echinococcosis (CE), a chronic disease caused by the larval form of the tapeworm *Echinococcus granulosus*, is one of the most important helminth-associated zoonoses globally [1], [2]. The parasite's domestic life cycle involves livestock and dogs as the primary intermediate and definitive hosts, respectively. Canids harboring adult *E. granulosus* worms excrete eggs into the environment, where intermediate hosts become infected through ingestion of the eggs. Humans can also act as aberrant intermediate hosts if they ingest infective parasite eggs either through contaminated food or directly from an infected canid. A cystic larval form (metacestode) gradually develops, most commonly in the liver or lungs. However, other organs can also be affected. Clinical signs typically develop as a result of this space-occupying lesion exerting pressure on surrounding tissues. Rupture of the cyst and spillage of the contents may cause anaphylactic shock and secondary CE. In many parts of the world, including Iran, surgery remains the treatment of choice for most individuals suffering from CE [2].

Cystic echinococcosis is a cosmopolitan zoonosis, with highly endemic areas especially prevalent in regions of South America, North Africa, China, and the Middle East [2]. Iran is an important endemic focus of CE where several species of intermediate host are commonly infected with *E. granulosus* [3]. High infection prevalences, with different strains of *E. granulosus*, have been reported in various domestic livestock including sheep (5.1%–74.4%), goats (2%–20%), cattle (3.5%–38.3%), buffalo (11.9%–70%), and camels (25.7%–59.3%) [4], [5], [6]. Between 5% and 45% of dogs is reported to be infected with *E.granulosus* in different provinces of Iran (reviewed in [7]). Human CE cases are also regularly reported from medical centers in different parts of the country and the incidence of CE is estimated 1.18-3 per 100,000 populations in different regions [7].

Recently, the World Health Organization (WHO) included CE in a subgroup of selected Neglected Tropical Diseases (NTDs) to be addressed within its 2008–2015 strategic plan for control of NTDs [8], [9]. The WHO recommends that the impact of zoonotic infections be assessed before implementation of any control measure [10], [11]. Costs associated with CE have been shown to have a great impact on affected individuals, their families, and the community as a whole [12], [13]. Monetary losses due to CE have been estimated for Uruguay [14], Wales [15], Jordan [16], Tunisia [17], Turkey [18], Spain [19], Peru [20] and for a highly endemic area of the Tibetan plateau [21], [22]. In addition, the non-monetary burden of CE has been assessed for a highly endemic region of China and globally utilizing the disability adjusted life year (DALY) [23].

Although CE is assumed to be a significant public health and economic problem in Iran, the extent of its socioeconomic impact is not fully understood. Economic losses due to CE in ruminants have been previously estimated in three provinces of Iran (Khuzestan, North Khorasan, and Ardabil) [24], [25], [26]. However, these studies were not concerned with human CE and used potentially biased methods to estimate livestock-related losses. Accurate assessment of the disease burden is crucial to raise awareness of decision-makers and to prioritize use of limited resources to provide timely preventive measures [27], [28]. The purpose of the present study is to estimate the monetary burden of CE in Iran using existing country-level data on human and animal CE.

## Materials and Methods

### Human epidemiological data

Population data for Iran for 2010 were extrapolated from the 2006 population census, with 71.8% of the population living in urban areas [29]. Due to a lack of surveillance data, the number of CE patients, by age and gender, that underwent surgery between 2000 and 2009 in 34 referral hospitals in seven of the country's most populous provinces (representing 51.4% of the total population) was collected to determine average annual surgical incidence. In total, 5,993 CE surgeries were identified over this 10-year period. For the remaining 23 provinces, data from individual scientific reports were used when available [30], [31], [32], [33]. For those provinces with no data, information from neighboring provinces with similar socioeconomic status was applied. Based on these sources, an annual number of 1,295 CE surgeries was calculated. All CE recurrences with re-operations were regarded as new surgical cases. Approximately 80% of surgical CE cases in Iran are treated in public hospitals, with the remaining 20% treated in private hospitals. Only surgical cases of CE were included in this study due to a lack of data on cases that seek treatment, but that are treated medically. In order to estimate the number of undiagnosed or asymptomatic cases of CE in Iran, data on ultrasound prevalence of CE (1.2% and 0.2%) were used (Table 1) [34], [35]. Lengths of hospital stay and mortality rates were based on available literature (Table 1).

**Table 1:** Human epidemiological parameters associated with CE in Iran.

*Category*	*Value*	*Unit*	*Distribution*	*Range*	*Reference*
Population (2010)	74,733,230	Individuals	Fixed	-	[29]
Urban/Rural	71.8/28.2	Percent	Fixed	-	[29]
Average income per day-urban	23.48	US$	Fixed	-	[29]
Average income per day-rural	14.09	US$	Fixed	-	[29]
Annual surgical incidence of CE	1.27	Per 100,000	Uniform	0.80–1.73	See Methods
Hepatic cysts	55.5	Percent	Fixed	-	See Methods
Pulmonary cysts	30.9	Percent	Fixed	-	[25], [31], [33], [51], [52]*
Hepatic and pulmonary involvement	4.1	Percent	Fixed	-	See Methods
Other organs	9.5	Percent	Fixed	-	See Methods
Undiagnosed cases of CE	0.85	Percent	Uniform	0.2–1.5	[34], [35]
Length of hospital stay	11.4	Days	Uniform	7–15.8	[53], [54], [55]
Mortality among surgical cases	2.5	Percent	Uniform	1–5	[56], [57], [58]
No of absentee days for recovery	18	Days	Uniform	8–28	[53], [54]
**Age and sex distribution** ¶	947	Individuals	Uniform	599–1295	See Methods
*Male patients*					
0–9	4.6	Percent	-	-	
10–19	14.5	Percent	-	-	
20–29	20.4	Percent	-	-	
30–39	16.9	Percent	-	-	
40–49	13.9	Percent	-	-	
50–59	10.5	Percent	-	-	
60–69	10.1	Percent	-	-	
70–79	7.5	Percent	-	-	
80+	1.6	Percent	-	-	
Total	100	Percent	-	-	
*Female patients*					
0–9	3.6	Percent	-	-	
10–19	9.7	Percent	-	-	
20–29	19.0	Percent	-	-	
30–39	19.2	Percent	-	-	
40–49	16.8	Percent	-	-	
50–59	13.9	Percent	-	-	
60–69	10.3	Percent	-	-	
70–79	6.3	Percent	-	-	
80+	1.2	Percent	-	-	
Total	100	Percent	-	-	

¶ Based on surgical incidence.

### Livestock epidemiological data

The livestock species primarily involved in the domestic cycle of CE in Iran are sheep, goats, cattle, buffalo, and camels. Data for livestock populations and annual numbers of slaughtered animals were obtained from official government reports (Table 2) [29]. The low percentage of the total sheep and goat population slaughtered annually (12.8% and 8.5%, respectively) may reflect the practice of slaughtering outside of abattoirs. To account for home slaughtering, losses were also evaluated assuming that slaughter rates are twice what are reported at the abattoirs, assuming a mean of 1.25 offspring per ewe/doe per year. Milk, wool, and hide/skin production values were based on either Statistical Center of Iran (SCI) reports or United Nation's Food and Agriculture Organization (FAO) FAOSTAT data [29], [36]. Livestock prevalence data were obtained from abattoir-based studies available from the literature. Only studies where a researcher confirmed the presence of CE cysts were included because abattoir-reported cases are not considered reliable in Iran. Prevalence data obtained from 3 or more studies were combined for cattle, sheep, and goats using a meta-analysis for proportions in *R* statistical data analysis software, ver. 2.12.0 (META package version 1.6-1; by Guido Schwarzer) (Table 2) [37]. Due to the limited available data for buffalos, a meta-analysis could not be performed for this species. Therefore, the mean prevalence from two studies on CE in buffalo in Iran (12.4% and 11.9%) was used [5], [26].

**Table 2:** Epidemiological parameters and annual livestock production values for Iran.

*Category*	*Value (CI)*	*Unit*	*Distribution*	*Reference*
**SHEEP**				
Population	49,976,138	Animals	Fixed	[29]
*No of slaughtered animals/year	6,446,354	Animals	Fixed	[29]
Prevalence of CE at abattoir	23.5 (8–39)	Percent	Normal	[5], [26], [59], [60], [61], [62], [63], [64], [65]
Meat production	390,000	Tonne	Fixed	[36]
Milk production	444,004	Tonne	Fixed	[29]
Skin/hide production	64,800	Tonne	Fixed	[36]
Wool production	52,455	Tonne	Fixed	[29]
**GOAT**				
Population	22,333,547	Animals	Fixed	[29]
*No of slaughtered animals/year	1,912,640	Animals	Fixed	[29]
Prevalence of CE at abattoir	8 (5–11)	Percent	Normal	[5], [26], [59], [60], [61], [62], [63], [64], [65]
Meat production	106,000	Tonne	Fixed	[36]
Milk production	270,157	Tonne	Fixed	[29]
Skin/hide production	18,875	Tonne	Fixed	[36]
Wool production	2,905	Tonne	Fixed	[29]
**CATTLE**				
Population	7,088,984	Animals	Fixed	[29]
No of slaughtered animals/year	1,432,270	Animals	Fixed	[29]
Prevalence of CE at abattoir	20 (13–27)	Percent	Normal	[5], [26], [59], [60], [61], [62], [63], [64], [65]
Meat production	360,000	Tonne	Fixed	[36]
Milk production	5,965,728	Tonne	Fixed	[29]
Hide/leather production	47,700	Tonne	Fixed	[36]
**BUFFALO**				
Population	191,438	Animals	Fixed	[29]
No of slaughtered animals/year	30,926	Animals	Fixed	[29]
Prevalence of CE at abattoir	12.5	Percent	Fixed	[5], [26]
Meat production	14,900	Tonne	Fixed	[36]
Milk production	245,000	Tonne	Fixed	[29]
Hide/leather production	2,048	Tonne	Fixed	[36]
**CAMEL**				
Population	151,932	Animals	Fixed	[29]
No of slaughtered animals/year	45,127	Animals	Fixed	[29]
Prevalence of CE at abattoir	32 (15–49)	Percent	Normal	[66], [67], [68], [69], [70], [71]
Meat production	1,680	Tonne	Fixed	[36]

* Assuming government-reported slaughter rates for sheep and goats.

### Human economic data

Costs associated with direct and indirect losses associated with human surgical CE were assessed. Direct costs included cost of surgery, hospital accommodation, diagnostic imaging, clinical laboratory and histopathology testing, and drug costs in both public and private hospitals. The Puncture Aspiration Injection Re-aspiration (PAIR) technique, which is widely used in other parts of the world, is rarely used in Iran. Therefore, the procedure was not costed in this study. Unit costs of services were obtained from official tariffs established by the Iranian Ministry of Health and Medical Education [38]. Service costs were calculated by multiplying the unit cost of an individual parameter by its frequency in the course of disease. Expert attending surgeons from Afzalipour Medical Center in Kerman, Iran were asked to estimate the frequency of common CE-associated procedures and services when these data were not available elsewhere.

Indirect costs associated with human CE included lost wages due to work absenteeism during hospitalization and recovery, due to time off to stay with a child with CE, and due to CE-related mortality. Income data for urban and rural populations were obtained from official reports of the CBI. Gender specific wage data were not available for Iran or its neighboring countries. Therefore, based on studies conducted in other regions, it was assumed that women earn approximately 0.70 times as much as men [19]. Breakdown of wages by age was also not available for Iran. Therefore, it was assumed that this breakdown would also be similar to the findings from other studies [19]. Unemployment figures were based on SCI data. Productivity for females who do not work outside of the home was assumed to be equivalent to 30% of the daily income of an officially employed female of the same age group [15].

A 100% loss of daily wages or productivity was assumed for CE surgical patients for the period of hospitalization. However, no losses were evaluated for unemployed patients since government unemployment benefits, which are received by all members of society whether they work in the public or private sector, were assumed to remain unchanged during the treatment period. Since unemployment benefit coverage is most likely not complete, the cost estimation is probably underestimated, especially in rural populations. For CE patients under the age of 18 years, a 30% wage loss for a man 30–39 years of age was applied for the period of hospitalization. This was based on the assumption that a parent would need to devote a proportion of his or her time to caring for the child [17], [19]. It was assumed that premature mortality causes an annual income loss of between 1 and 364 days in any given year. Therefore, a uniform distribution was defined for the number of lost days due to CE-related deaths. In asymptomatic individuals, lost wages were calculated in terms of annual monetary income and a productivity loss of 0–5% for one year (Table 1).

### Livestock economic data

Direct and indirect costs due to CE-associated losses in livestock species were evaluated. Direct costs associated with CE in livestock are due to the condemnation of livers and lungs during carcass inspections in abattoirs. A uniform distribution was applied to liver and lungs losses based on market prices across Iran (Table 3). It was assumed that the entire liver and/or lungs of infected cattle, sheep, goats, and buffalo would be condemned. The cost of infected camel livers, but not lungs, was included in the estimate because camel lungs are not traditionally consumed in Iran.

**Table 3:** Value of livestock parameters (per Kg) used to estimate the monetary burden of CE in Iran.

*Category*	*Value (US$)*	*Distribution*	*Range*	*Reference*
**SHEEP**				
Live animal	2.86	Uniform	2.46–3.26	[29], [36]
Liver	10.12	Uniform	8.67–11.56	[72]
Lung	10.12	Uniform	8.67–11.56	[72]
Milk	0.50	Fixed	-	[36]
Hide/skin	1.64	Fixed	-	[73]
Wool	0.59	Fixed	-	[29]
**GOAT**				
Live animal	2.78	Uniform	2.40–3.16	[29], [36]
Liver	10.12	Uniform	8.67–11.56	[72]
Lung	10.12	Uniform	8.67–11.56	[72]
Milk	0.48	Fixed	-	[36]
Hide/skin	1.14	Uniform	0.63–1.64	[73]
Wool	0.59	Fixed	-	[29]
**CATTLE**				
Live animal	2.54	Uniform	2.26–2.81	[29], [36]
Liver	8.67	Uniform	7.71–9.63	[72]
Lung	0.27	Uniform	0.24–0.29	[72]
Milk	0.38	Fixed	-	[36]
Hide/leather	1.14	Uniform	0.63–1.64	[73]
**BUFFALO**				
Live animal	2.54	Uniform	2.26–2.81	[29], [36]
Liver	8.67	Uniform	7.71–9.63	[72]
Lung	0.27	Uniform	0.24–0.29	[72]
Milk	0.51	Fixed	-	[36]
Hide/leather	1.14	Uniform	0.63–1.64	[73]
**CAMEL**				
Live animal	1.21	Fixed	-	[36]
Liver	8.67	Uniform	7.71–9.63	[72]*
Milk	0.38	Fixed	-	[36]*

• Assumed to be similar to that of cattle.

Indirect losses due to decreased carcass weight, reduction in milk production, decreased wool production, decreased hide/skin value, and reproductive losses were estimated. Values of livestock parameters used to estimate economic losses associated with CE were assumed to be similar to those used in previous assessments of livestock-associated CE losses [16], [17], [19], [21]. Based on these values, a 2.5% decrease in milk production, 15% reduction in wool quality, 5.5% reduction in fecundity, 10% decrease in hide/skin production, and 6.25% reduction in carcass weight were utilized for this study. Farmers' investment was not taken into account in the presented cost estimates due to lack of data on this topic available from Iran or other countries in this region.

### Uncertainty and sensitivity analysis

Data were compiled in Excel spreadsheets (Microsoft Corp, Redmond, WA). The risk analysis and simulation software @RISK (Palisade corp., Ithaca, NY, ver. 4.5) for Excel was used to estimate monetary costs attributed to CE infection in humans and livestock. Output variables were defined according to parameters involved in the estimation of direct and indirect costs associated with CE in humans and livestock intermediate hosts (Table 4). Distributions were assigned based on the most likely range for each variable. Median and 2.5 and 97.5 percentiles (95% credible intervals, CIs) were calculated for each output variable. Monte Carlo simulation using a Latin Hypercube approach with 10,000 iterations was performed to model parameter uncertainty. A sensitivity analysis was conducted using stepwise linear regression of the estimated costs against the input parameter values to assess the impact of each input parameter on the overall cost estimate. A separate sensitivity analysis was run excluding losses related to asymptomatic/non-healthcare seeking human CE cases.

**Table 4:** Annual direct and indirect costs associated with CE in humans and livestock in Iran.

*Category*	*Median cost (US $)*	*95% CI*
**HUMAN**		
Costs of hepatic CE	593,485	410,640–818,157
Costs of pulmonary CE	261,800	189,390–340,775
Costs of CE in liver and lung	75,420	53,198–100,919
Costs of CE in other organs	101,456	70,080–139,730
Direct costs of CE	1,097,950	855,548–1,381,656
Indirect costs of CE§	372,613	188,873–576,448
Indirect costs of CE¶	97,527,670	9,712,122–206,574,100
Total costs of human CE§	1,470,564	1,158,458–1,817,444
Total costs of human CE¶	98,625,620	10,739,470–207,912,300
* **SHEEP**		
Direct costs of CE	12,524,960	4,047,542–22,354,430
Indirect costs of CE	59,036,660	4,047,542–22,354,430
Total costs of sheep CE	71,551,620	16,585,770–152,227,400
* **GOAT**		
Direct costs of CE	1,074,601	608,845–1,610,484
Indirect costs of CE	6,031,210	1,271,019–12,306,230
Total costs of goat CE	7,105,811	2,235,714–13,586,770
**CATTLE**		
Direct costs of CE	9,992,240	6,412,567–13,777,960
Indirect costs of CE	47,920,830	18,608,570–84,215,220
Total costs of cattle CE	57,913,070	27,012,570–96,117,080
**BUFFALO**		
Direct costs of CE	131,108	114,636–148,589
Indirect costs of CE	787,311	314,762–1,273,489
Total costs of buffalo CE	918,418	445,066–1,403,014
**CAMEL**		
Direct costs of CE	13,433	6,201–20,886
Indirect costs of CE	586,974	175,347–1,140,535
Total costs of camel CE	600,406	184,034–1,158,064
**All animals**		
Direct costs	23,726,340	14,323,200–34,387,130
Indirect costs	114,363,000	51,049,920–196,475,100
Total costs of animal CE	138,089,300	69,524,500–226,669,800
**Direct costs of CE in human and animals**	24,824,290	15,425,180–35,444,500
**Indirect costs of CE in human and animals** §	114,735,600	51,377,930–196,922,400
**Indirect costs of CE in human and animals** ¶	211,890,700	96,003,140–344,185,200
**TOTAL MONETORY COSTS OF CE IN IRAN** §	**139,559,900**	**71,095,360–228,152,000**
**TOTAL MONETORY COSTS OF CE IN IRAN** ¶	**236,714,900**	**117,690,300–373,694,500**

* Assuming government-reported slaughter rates for sheep and goats.

§ Excluding asymptomatic/non-healthcare seeking human population.

¶ Including asymptomatic/non-healthcare seeking human population.

## Results

### Human CE costs

Table 4 contains estimates of the annual direct and indirect costs associated with CE in humans in Iran. The cost of surgical treatment for a case of hepatic or pulmonary CE in a public hospital was estimated at US$1,027 (95% CI US$676–1,379) and US$851 (95% CI US$528–1,173), respectively. The corresponding values for surgical treatment of CE in a private hospital were estimated at US$1,911 (95% CI US$1,431–2,387) for hepatic and US$2,458 (95% CI US$1,976–2,939) for pulmonary involvement. The overall annual cost of CE in Iran was estimated at US$232.25 million (95% CI US$103.11–397.84 million). The cost associated with human CE was estimated at US$93.39 million (95% CI US$6.11–222.72 million), of which US$1.09 million (95% CI US$820,000–1.44 million) and US$92.34 million (95% CI US$5.01–221.55 million) were attributed to direct and indirect costs, respectively.

Human CE contributed to more than 40% of the total annual cost of CE in Iran. This was mainly due to the impact of human productivity losses in the asymptomatic/non-healthcare seeking population. This figure decreased to 1.1% of the total estimated cost when productivity losses in the asymptomatic/non-healthcare seeking population were excluded. Direct costs of human CE were estimated at 1.2% of the total cost of human disease. However, direct costs accounted for three quarters of the economic losses in surgical CE cases.

### Livestock associated CE costs

Assuming government slaughter values, the median annual cost associated with CE in livestock was estimated at US$132.0 million (95% CI US$61.8–246.5 million), of which US$23.5 million (95% CI US$12.7–36.5 million) was direct and US$108.4 million (95% CI US$45.0–216.9 million) was indirect cost. Sheep and cattle CE were responsible for 48% and 42% of the total economic losses due to livestock CE in Iran, respectively. Direct costs associated with CE in livestock accounted for 10.1% of the overall cost of the disease. Indirect costs associated with CE in livestock were primarily due to losses in fecundity and milk reduction. Indirect costs due to CE in livestock intermediate hosts comprised more than 80% of the total livestock-associated costs of CE and approximately 47% of the overall cost of CE in Iran. Costs associated with sheep and goat CE, assuming the practice of home slaughtering, are found in Table 5.

**Table 5:** Estimated monetary losses associated with CE in Iran based on two scenarios for home slaughtering.

Scenarios		Direct costs, US$ (95% CI)	Indirect costs, US$ (95% CI)	Direct and indirect costs, US$ (95% CI)
**Government reported values assuming that 12.8% of sheep and 8.5% of goats are slaughtered annually.**	Sheep	12,524,960 (4,047,542–22,354,430)	59,036,660 (9,746,846–133,468,100)	71,551,620 (16,585,770–152,227,400)
	Goat	1,074,601 (608,845–1,610,484)	6,031,210 (1,271,019–12,306,230)	7,105,811 (2,235,714–13,586,770)
	**Total**	**13,589,560 (5,037,907–23,589,440)**	**65,067,870 (15,316,950–139,935,100)**	**78,657,420 (22,988,640–159,876,500)**
**Adjusting for home slaughtering assuming that 25% of sheep and 17% of goats are slaughtered annually**.	Sheep	25,048,910 (8,168,014–44,242,900)	63,456,400 (12,570,030–140,369,500)	88,505,300 (24,126,530–177,011,800)
	Goat	2,149,359 (1,225,908–3,259,446)	6,400,930 (1,580,594–12,635,600)	8,550,289 (3,359,383–15,273,460)
	**Total**	**27,198,270 (10,138,330–46,741,520)**	**69,857,330 (18,569,920–146,739,300)**	**97,055,590 (32,653,080–186,131,500)**

### Sensitivity analysis

**Figure 1:**
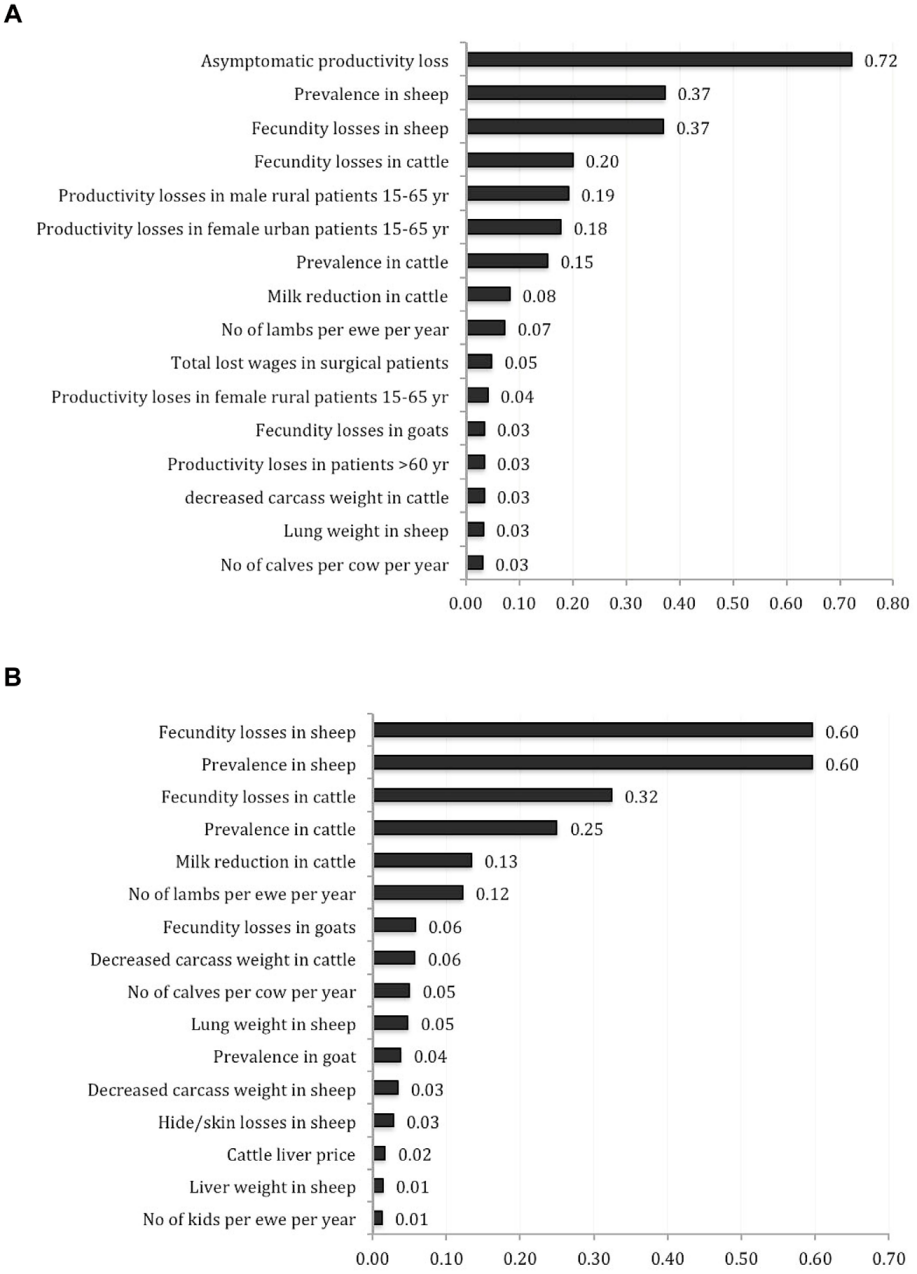
Regression coefficients of parameters associated with the total cost of CE in Iran.

The impact of uncertain parameters on the total monetary burden of CE in Iran and the corresponding regression coefficient values are shown in Figure 1. Productivity losses in asymptomatic individuals, CE prevalence in sheep, and fecundity losses in sheep and cattle had the largest impact on overall cost of the disease (Figure 1a). When productivity losses in asymptomatic/non-healthcare seeking individuals were excluded, fecundity losses and CE prevalence in sheep and cattle had the largest overall impact (Figure 1b).

## Discussion

Estimating the economic impact of a zoonotic disease is a way of quantifying the significance of the disease in both human and livestock populations. In addition, this type of analysis helps decision-makers prioritize resources for disease control and prevention. The aim of the present study was to estimate the economic impacts of CE in Iran. Findings indicated that CE costs Iran more than US$230 million per year. This is a considerable burden as this equates to about 0.03% of the country's Gross Domestic Product (GDP). A value of 0.03% of the country's GDP is in line with the findings of other studies where this value ranged from 0.003% to 0.04% of GDP (Table 6).

**Table 6:** Human and livestock population and the proportion of GDP lost due to CE in different countries.

*Country*	*Human population (million humans)*	*Livestock population (million animals)*			*Total cost of CE (US$ million)*	*% GDP lost*	*Reference*
		*Sheep*	*Goat*	*Cattle*			
Iran	74.7	49.9	22.3	7.1	232.3	0.03	Present study
Spain	43.0	22.7	2.9	6.5	200	0.01	[19]
Turkey	74.7	25.5	6.3	11.0	89¶	0.01¶	[18]
Tunisia	9.6	7.2§	1.3	0.8	14.7	0.03	[17]
Uruguay	3.2	25.0	0.016§	10.4	9.0	0.04	[14]
Peru	26.2	14.2§	1.9§	5.5§	6.3	0.003*	[20]
Jordan	4.4	1.2	0.5	0.7§	3.9	0.04	[16]

¶ Cost of animal CE only.

§ From FAOSTAT, 2010.

* Indirect human losses not accounted for.

The overall cost of CE in Iran was estimated to be higher than the CE-associated monetary losses for other countries, including Jordan (US$ 3.9 million), Uruguay (US$ 9.0 million), Tunisia (US$ 14.7 million), Turkey (US$ 89 million- livestock losses only), and Spain (US$ 200 million) [14], [15], [16], [18], [19]. This is partly the result of larger human and livestock populations in Iran compared to the other studied countries (Table 6). Iran is the third most populous and second largest country in the Middle East and has the fourth largest sheep population in the world [36]. However, direct comparison of economic losses associated with CE from different countries is difficult since past studies have used a variety of methodologies to arrive at cost estimates.

In previous studies on ruminant echinococcosis economic losses due to CE have been estimated using conventional calculation methods. Livestock CE-related losses were estimated at US$459,660 in the city of Ahwaz [25], at US$421,826 in nine districts of North Khorasan province [24] and at US$51,900 in Ardabil province [26].

Based on the results of this study, the monetary burden of CE in Iran is substantial, especially when indirect costs due to productivity losses in the asymptomatic/non-healthcare seeking population were taken into consideration. Productivity losses for asymptomatic/non-healthcare seeking individuals added about US$ 100 million to the overall cost estimate of CE in the country. This estimate was based on the two community-based ultrasound studies that have been carried out in Iran. However, this was not optimal since both of the studies were conducted in rural/nomadic populations. Nevertheless CE cases are increasingly reported from urban regions. The number of CE cases from rural and urban areas was shown not be significantly different in Iran. Several studies have shown that CE is equally prevalent in rural and urban regions, especially due to the increased recreational/camping activities of the urban population and large migrations of people from rural to the urban/peri-urban regions of the country during last three decades [31], [39]. This same phenomenon has been documented in other countries, including Serbia [40], Croatia [41] and Libya [42]. Like other NTDs prevalent in less developed countries, it appears that CE is being urbanized and can no longer be considered solely as a rural disease [43], [44].

The ratio of community ultrasound prevalence to the annual surgical incidence of CE was 669.3, which is higher than the ratio of 45.4 found in Florida, Uruguay [45], the ratio of 22 to 344 for Turkey [46], [47], and the ratio of 241 for Morocco [47]. However, the value is comparable with the ratio of 405 to 1,889 determined for Libya [47], [48]. While this may mean that the number of asymptomatic/non-healthcare seeking individuals in Iran was overestimated, it also could indicate that health-seeking behavior of Iranians is different from that of people in other countries. Compared to Uruguayans, Turks, and Moroccans, Iranians may have either less access to health care or do not seek health care services provided in the country due to different health-seeking behaviors. Differences in the pathogenicity of *E. granulosus* genotypes/strains may also explain this dissimilarity since it is generally believed that genotypes of *E. granulosus* can differ in infectivity and/or clinical severity [49]. By applying the ratios for Turkey (334) and Uruguay (45.4) to the incidence rate of surgical cases in Iran, the prevalence of asymptomatic/non-healthcare seeking cases of CE would be 0.23% and 0.06%, respectively compared to the estimated 0.85% used in this study.

A limitation of this study was how to assess productivity losses for those individuals who were not formally employed. Based on limited available data, a 30% productivity loss was assumed for women who are not officially employed outside of the home. This value was chosen because a sick homemaker indirectly affects the entire family's productivity and increases living costs of the family. Indirect costs of CE in humans and livestock accounted for more than 80% of overall monetary losses in this study, which is in agreement with the results of other studies in endemic areas [14], [15], [16], [17], [18], [19], [21]. Indirect costs reflect economic effects of the disease that are often not taken into consideration by agriculture and health officials. Indirect costs associated with human CE treatment were probably underestimated in this study. Additional indirect costs may include expenses associated with travel from a rural area to the city, or from one urban area to another urban area to seek appropriate health care, as well as expenses due to an accompanying spouse or other member of the family. Additional studies are needed in order to provide better evidences of the true impact of indirect losses due to CE in both humans and livestock intermediate hosts.

Availability of high quality epidemiological and economic data is crucial for improving the accuracy of the estimation. Lack of age-stratified CE prevalence data for livestock was another limitation of the present study. However, abattoir-based CE prevalence data tends to be underestimated due to the fact that, in Iran, animals that are slaughtered in abattoirs tend to be young and, therefore, have a lower chance of being infected compared to older animals. Another important issue is the unexpectedly low proportion of the sheep and goat population reported to be slaughtered each year (12.8% and 8.5%, respectively). These figures reflect animals that are slaughtered in registered abattoirs, which is almost definitely an underestimation. Many people, especially those living in rural/suburban areas, practice home slaughter. In addition, a number of unregulated abattoirs also exist within the country. However, the extent of slaughtering outside official channels is not fully understood and needs to be investigated. To account for the practice of home slaughter, a second scenario was considered assuming that 25% and 17% of sheep and goat populations are slaughtered every year, respectively. As expected, this second scenario resulted in both increased direct and indirect costs for these species (Table 5). However, the overall effect of the second scenario on the total monetary cost of human and animal CE was relatively small (i.e., a 7.7% increase from US$236.7 million to US$254.9 million). Regarding the high proportion of camel population reported to be slaughtered each year (29.7%) that seems very high for such a long-lived animal, we retrieved camel data from official sources (Statistical Center of Iran). Underestimation of the total population of camels is quite probable because of the very traditional nature of camel farming in the country and illegal import of camels across the eastern border.

Findings of the present study indicate that CE imposes a substantial economic impact on Iran. Reduction of human and livestock infection through implementation of CE control programs is necessary to reduce the economic burden of CE on the country. Cost-benefit analysis of different control programs is now possible in light of present knowledge on the economic losses associated with CE in Iran. However, because comparing monetary costs in different countries with different socioeconomic statuses is often not optimal, a complementary analysis of the non-monetary burden of CE is recommended to compare CE burden in different geographical regions. In addition, evaluation of the non-monetary burden of the disease and measurement of cost per DALY averted by the control campaigns is recommended. Therefore, a paper evaluating CE-associated DALYs in Iran is currently in preparation. This is the first study to evaluate monetary losses due to human and livestock CE in Iran. However, additional research is needed to improve CE monetary burden estimates and to develop uniform methodologies for assessment [17], [50].

## Supporting Information

Checklist S1 STROBE checklist.
(DOC)
