# Supplementary material for: The Monetary Burden of Cystic Echinococcosis in Iran
Source: PLoS Negl Trop Dis. 2012 Nov 29;6(11):e1915. doi: 10.1371/journal.pntd.0001915 (PMC3510083; doi:10.1371/journal.pntd.0001915)
Supplement: Checklist S1 — STROBE checklist. (DOC) [file pntd.0001915.s001.doc]

STROBE Statement—Checklist of items that should be included in reports of ***cross-sectional studies***

|  | Item No | Recommendation |
| --- | --- | --- |
| **Title and abstract** | ✓1 | (*a*) Indicate the study’s design with a commonly used term in the title or the abstract. **The design is indicated in the abstract.** |
|  |  | (*b*) Provide in the abstract an informative and balanced summary of what was done and what was found. **The summary is provided in the abstract.** |
| Introduction | | |
| Background/rationale | ✓2 | Explain the scientific background and rationale for the investigation being reported. **The background is explained in the Introduction.** |
| Objectives | ✓3 | State specific objectives, including any prespecified hypotheses. **The objectives are included in the Introduction.** |
| Methods | | |
| Study design | ✓4 | Present key elements of study design early in the paper. **Details of study design are provided in the Methods.** |
| Setting | ✓5 | Describe the setting, locations, and relevant dates, including periods of recruitment, exposure, follow-up, and data collection. **The settings of the study are described in detail.** |
| Participants | ✓6 | (*a*) Give the eligibility criteria, and the sources and methods of selection of participants. **Not applicable.** |
| Variables | ✓7 | Clearly define all outcomes, exposures, predictors, potential confounders, and effect modifiers. Give diagnostic criteria, if applicable. **Not applicable.** |
| Data sources/ measurement | ✓8* | For each variable of interest, give sources of data and details of methods of assessment (measurement). Describe comparability of assessment methods if there is more than one group. **Data sources are described in the Methods.** |
| Bias | ✓9 | Describe any efforts to address potential sources of bias. **The sources of bias are described.** |
| Study size | ✓10 | Explain how the study size was arrived at. **The method for study size is explained.** |
| Quantitative variables | ✓11 | Explain how quantitative variables were handled in the analyses. If applicable, describe which groupings were chosen and why. **Analyses methods for quantitative variables are described.** |
| Statistical methods | ✓12 | (*a*) Describe all statistical methods, including those used to control for confounding |
|  |  | (*b*) Describe any methods used to examine subgroups and interactions |
|  |  | (*c*) Explain how missing data were addressed |
|  |  | (*d*) If applicable, describe analytical methods taking account of sampling strategy |
|  |  | (*e*) Describe any sensitivity analyses. **Statistical methods are described in details.** |
| Results | | |
| Participants | ✓13* | (a) Report numbers of individuals at each stage of study—eg numbers potentially eligible, examined for eligibility, confirmed eligible, included in the study, completing follow-up, and analysed. **Not applicable.** |
|  |  | (b) Give reasons for non-participation at each stage. **Not applicable.** |
|  |  | (c) Consider use of a flow diagram |
| Descriptive data | ✓14* | (a) Give characteristics of study participants (eg demographic, clinical, social) and information on exposures and potential confounders. **Not applicable.** |
|  |  | (b) Indicate number of participants with missing data for each variable of interest. **Not applicable.** |
| Outcome data | ✓15* | Report numbers of outcome events or summary measures. **Outcome data are explained.** |
| Main results | ✓16 | (*a*) Give unadjusted estimates and, if applicable, confounder-adjusted estimates and their precision (eg, 95% confidence interval). Make clear which confounders were adjusted for and why they were included. **The estimates as well as credible intervals are included in the results.** |
|  |  | (*b*) Report category boundaries when continuous variables were categorized |
|  |  | (*c*) If relevant, consider translating estimates of relative risk into absolute risk for a meaningful time period. **Not applicable.** |
| Other analyses | ✓17 | Report other analyses done—eg analyses of subgroups and interactions, and sensitivity analyses. **Details of sensitivity analyses are provided.** |
| Discussion | | |
| Key results | ✓18 | Summarise key results with reference to study objectives. **Key results are provided in the Discussion.** |
| Limitations | ✓19 | Discuss limitations of the study, taking into account sources of potential bias or imprecision. Discuss both direction and magnitude of any potential bias. **Limitations of the study are discussed in detail.** |
| Interpretation | ✓20 | Give a cautious overall interpretation of results considering objectives, limitations, multiplicity of analyses, results from similar studies, and other relevant evidence. **Done.** |
| Generalisability | ✓21 | Discuss the generalisability (external validity) of the study results. **Done.** |
| Other information | | |
| Funding | ✓22 | Give the source of funding and the role of the funders for the present study and, if applicable, for the original study on which the present article is based. **Described in the submission process.** |

*Give information separately for exposed and unexposed groups.

**Note:** An Explanation and Elaboration article discusses each checklist item and gives methodological background and published examples of transparent reporting. The STROBE checklist is best used in conjunction with this article (freely available on the Web sites of PLoS Medicine at http://www.plosmedicine.org/, Annals of Internal Medicine at http://www.annals.org/, and Epidemiology at http://www.epidem.com/). Information on the STROBE Initiative is available at www.strobe-statement.org.
